# X-linked hyper-IgM syndrome with eosinophilia in a male child: A case report

**DOI:** 10.3892/etm.2015.2261

**Published:** 2015-02-05

**Authors:** LI GUO, BO CHEN, BIN XU, MEIPING LU, BOTAO NING, ZHENJIE CHEN

**Affiliations:** 1 Department of Rheumatology, Immunology and Allergy, Children’s Hospital, Zhejiang University School of Medicine, Hangzhou, Zhejiang 310003, P.R. China; 2 Department of Children’s Rehabilitation, Zhejiang Armed Police Hospital, Hangzhou, Zhejiang 310003, P.R. China; 3 Department of ENT, Children’s Hospital, Zhejiang University School of Medicine, Hangzhou, Zhejiang 310003, P.R. China; 4 Department of PICU, Children’s Hospital, Zhejiang University School of Medicine, Hangzhou, Zhejiang 310003, P.R. China

**Keywords:** hyper-IgM syndromes, CD40/CD40L signaling pathway, eosinophilia

## Abstract

The hyper-IgM syndromes (HIGMs) are a group of primary immune deficiency diseases characterized by a normal or elevated serum level of IgM and low or absent serum levels of IgG, IgA and IgE. Here, we report a case of X-linked HIGM with a new CD40L gene mutation presenting with eosinophilia. The patient experienced recurrent pneumonia and acute respiratory distress syndrome (ARDS) from 4 months of age. Immunological evaluation revealed a normal level of serum IgM, with significantly low levels of serum IgG and IgA. Genetic analysis of the CD40L gene revealed a splice mutation in exon 5 at the nucleotide position 410 (c.410-2A>G), which has never been reported previously in the literature. Following treatment with regular intravenous immunoglobulin (IVIG) replacement therapy every 3 to 4 weeks and infection prophylaxis with trimethoprim-sulfamethoxazole during follow-up, the patient’s immunoglobulin level returned to normal with no pulmonary infection. The eosinophil count also returned to normal after a small dose of steroid agent treatment was administered orally for 5 months. In summary, X-linked hyper-IgM syndrome with CD40L gene mutation presenting with eosinophilia may be successfully treated using IVIG replacement therapy and a small dose of steroid agent.

## Introduction

The hyper-IgM syndromes (HIGMs) are a group of rare primary immune deficiency diseases characterized by a normal or elevated serum level of IgM and low or absent serum levels of IgG, IgA and IgE with normal peripheral blood B lymphocyte counts (1). The mechanism of HIGM is immunoglobulin class-switch recombination (CSR) failure and somatic hyper mutation (SHM), which is caused by molecular defects in the CD40L/CD40 signaling pathway or defects involving enzymes required for CSR and SHM (2). The X-linked form of HIGM is caused by either the CD40L gene mutation or NF-κB essential modulator defects (3). To date, eight genetically defined forms of HIGM have been documented, of which HIGM1 is the most common and the X-linked form, caused by CD40L gene mutations (4).

Patients with HIGM are susceptible to recurrent sino-pulmonary infections, neutropenia, autoimmune diseases and malignancies, and opportunistic infections including pneumocystis carinii pneumonia (PCP) and cryptosporidium (1,2). In the treatment of patients with HIGM, immunoglobulin (Ig) replacement can reduce the frequency and severity of infections, but cannot prevent malignancies. Infections should be treated aggressively with specific antimicrobial therapy and cases of PCP require prophylaxis with oral trimethoprim-sulfamethoxazole. The ideal length of prophylaxis remains unknown. Neutropenia can be successfully treated with granulocyte-colony stimulating factor. At present, stem cell transplantation remains the most effective method of HIGM treatment. Genetic therapy for HIGM is currently in the experimental stages (1). In the present study, we report a case of X-linked HIGM with a new CD40L gene mutation (HIGM1) presenting with eosinophilia in a young Chinese boy.

## Case report

### Case presentation

**Figure 1:**
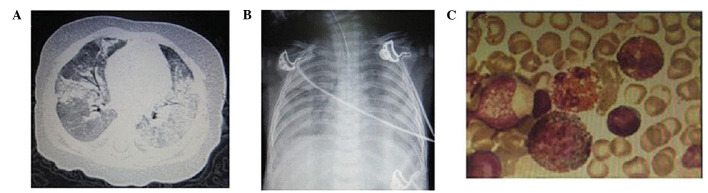
Clinical examination. (A) Chest computed tomography scan revealing extensive consolidation on both sides of the lung; (B) Chest X-ray revealing acute respiratory distress syndrome; (C) Bone marrow examination revealing additional eosinophils.

A 1-year-old boy had been developing normally from birth to 3 months old. However, he presented recurrent pulmonary infection from the age of 4 months. The patient was admitted to the Children’s Hospital of Zhejiang University School of Medicine (Hangzhou, China) twice due to acute respiratory distress syndrome (ARDS; Fig. 1A and B) at 4 and 7 months old of age, respectively. The present study was approved by the Ethics Committee of the Children’s Hospital of the Zhejiang University School of Medicine (Hangzhou, China). Written informed consent was obtained from the patient’s family prior to participation.

### Diagnosis

**Figure 2:**
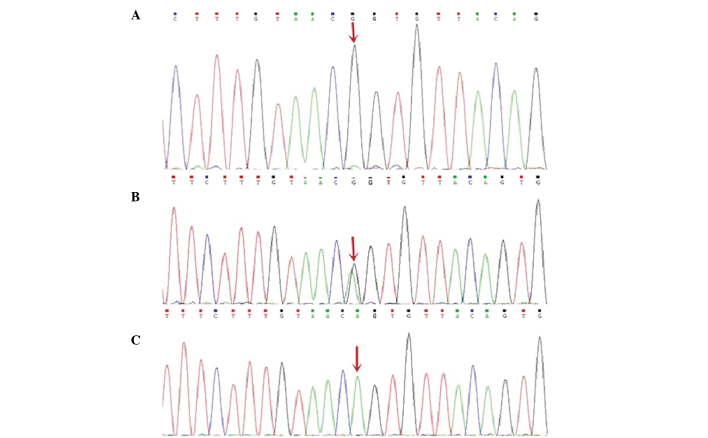
Gene analysis of CD40L in the patient and his parents. (A) Patient: A to G substitution in exon 5 (c.410-2A>G); (B) Patient’s mother: A to G heterozygous mutation in exon 5 (c.410-2A>G); (C) Patient’s father: no mutation in exon 5 (c.410-2A>G).

Blood tests revealed a significantly increased white blood cell count with eosinophilia (18–25%); however, neutrophil counts were within the normal range. Bone marrow aspiration (Fig. 1C) revealed an increased proportion of eosinophils (23.5%) with no morphological evidence of dysplasia. An investigation of pathogens, including tuberculosis, parasites, atypical pathogens and viruses, revealed no abnormalities. The ARDS condition was improved and the patient’s eosinophil count was quickly reduced to the normal range with the support of high-frequency oscillator ventilation and co-treatment of antibiotics and glucocorticoids. However, the recurrent pulmonary infections remained. Further examinations revealed that serum immunoglobulin levels of IgG (0.16 g/l) and IgA (0.01 g/l) were significantly reduced; however, the IgM (0.86 g/l) level was within the normal range. No abnormal lymphocyte subsets were identified. Gene sequencing analysis of the CD40L gene revealed a homozygous G to A substitution in exon 5 (c.410-2A>G; Fig. 2). Parental DNA analysis revealed that the patient’s mother was a carrier. The diagnosis of X-linked HIGM was confirmed.

### Treatment

Following treatment of intravenous immunoglobulin (IVIG), trimethoprim-sulfamethoxazole and glucocorticoid agents, the pulmonary infection was significantly reduced. The oral glucocorticoid agent was discontinued once the eosinophil count had remained within normal range for 5 months. During the follow-up period, the patient received regular IVIG replacement therapy every 3 to 4 weeks and intermittent infection prophylaxis with trimethoprim-sulfamethoxazole. The patient is currently well developed with normal immunoglobulin levels.

## Discussion

HIGM1 is the most common HIGM phenotype and accounts for approximately 65–70% of all cases. HIGM1 patients usually have an onset of symptoms before 2 years of age, presenting as recurrent sino-pulmonary infections. The majority of patients are susceptible to PCP and cryptosporidium associated with diarrhea, sclerosing cholangitis and tumors of the liver, pancreas or biliary tract (5,6). Autoimmune diseases and neutropenia associated with stomatitis are relatively common (5,7). HIGM1 patients require repeated IVIG replacement treatment at doses of 400–600 mg/kg every 3 to 4 weeks and should receive prophylaxis of trimethoprim-sulfamethoxazole for PCP. Stem cell transplantation may cure X-linked HIGM as well as the associated neutropenia, cholangiopathy and liver failure, in combination with a liver transplant (8).

The CD40L gene is located in the long arm of the X-chromosome (Xq26-27) and includes five exons. CD40L is a 39-kDa type II membrane glycoprotein belonging to the superfamily of tumor necrosis factors and is expressed on the surface of the activated CD4^+^ T cells. CD40L is crucial for T-B cell interaction by binding to CD40, which is expressed in B cells. CD40L gene mutation results in the reduced expression of CD40L in CD4^+^ T cells, which interferes with the interaction of CD40L and CD40 or influences the formation of CD40 trimer molecules (7,9). Therefore, the CD40L gene defect destroys the T-B cell interaction and affects CSR. At present, 250 unique mutations of the CD40L gene have been identified (http://bioinf.uta.fi/CD40Lbase), mainly in exon 5 but also in exon 4 (7).

Our patient suffered recurrent pneumonia and ARDS. Although no evidence of PCP was observed, his pulmonary infection was significantly reduced following trimethoprim-sulfamethoxazole treatment. Eosinophilia was a prominent clinical manifestation in the patient. To date, there there has only been one report of eosinophilia in patients with HIGM1 (10), and the pathogenesis of HIGM1 complicated with eosinophilia remains unclear. Neutropenia did not exist in the patient. Immunological evaluation revealed a normal level of serum IgM, significantly low levels of serum IgG and IgA, and normal counts of peripheral blood B cells. Sequencing analysis of the CD40L gene revealed a splice mutation within exon 5 at nucleotide position 410 (c.410-2A>G), which has never been reported previously in the literature. After the patient received regular IVIG replacement therapy at 3 to 4 weeks intervals and intermittent trimethoprim-sulfamethoxazole prophylaxis for PCP, his immunoglobulin levels returned to normal with no further recurrent pulmonary infection. The boy is currently developing normally.
